# Mesenchymal stromal cells for acute graft‐versus‐host disease: response at 1 week predicts probability of survival

**DOI:** 10.1111/bjh.15749

**Published:** 2019-01-13

**Authors:** Antonio Galleu, Dragana Milojkovic, Simona Deplano, Richard Szydlo, Sandra Loaiza, Robert Wynn, David I. Marks, Deborah Richardson, Kim Orchard, Edward Kanfer, Eleni Tholouli, Muhammad Saif, Ponni Sivaprakasam, Sarah Lawson, Adrian Bloor, Antonio Pagliuca, Victoria Potter, Varun Mehra, John A. Snowden, Ajay Vora, Bhuvan Kishore, Hannah Hunter, Jane F. Apperley, Francesco Dazzi

**Affiliations:** ^1^ King's College London London UK; ^2^ King's Health Partners Cancer Research UK Centre London UK; ^3^ Imperial College Healthcare NHS Trust London UK; ^4^ Imperial College London London UK; ^5^ Central Manchester University Hospital Manchester UK; ^6^ University Hospitals Bristol Bristol UK; ^7^ University Hospital Southampton Southampton UK; ^8^ Birmingham Women's and Children's Hospitals Birmingham UK; ^9^ The Christie NHS Foundation Trust Manchester UK; ^10^ King's College Hospital NHS Trust London UK; ^11^ Sheffield Teaching Hospitals NHS Foundation Trust Sheffield UK; ^12^ Sheffield Children's Hospital Sheffield UK; ^13^ Heart of England NHS Foundation Trust Birmingham UK; ^14^ Plymouth Hospitals NHS Trust Plymouth UK

**Keywords:** cellular therapies, GvHD, mesenchymal cells, clinical research, stromal cells

## Abstract

Mesenchymal stromal cells (MSCs) have been successfully used for the treatment of steroid‐resistant graft‐versus‐host‐disease (GvHD). However, the lack of early predictors of clinical responses impacts on the time at which to add further treatment and consequently the design of informative clinical trials. Here, we present the UK experience of one of the largest cohorts of GvHD patients undergoing MSC infusions so far reported. We show that clinical responses assessed as early as 1 week after MSC infusion predict patients’ overall survival. In our cohort, cell dose, patients’ age and type of organ involvement are crucial factors associated with clinical responses.

Acute graft‐versus‐host disease (aGvHD) is a life‐threatening complication of allogeneic haematopoietic stem cell transplantation (HSCT) and one of the major factors limiting its success (Ferrara *et al*, 2009). Currently there is no established strategy to treat steroid‐resistance in aGvHD patients, which is associated with a dismal prognosis. Mesenchymal stromal cells (MSCs) are a heterogeneous population of cells with potent immunosuppressive activity (Marigo & Dazzi, 2011). They have been extensively tested as a salvage option to treat steroid‐resistant aGvHD patients and convincingly shown to improve the survival of responding patients (Le Blanc *et al*, 2008; von Dalowski *et al*, 2016). Despite these encouraging results, there is still a pressing need to identify reliable factors that can be used as early predictors of treatment outcome, to identify those patients more likely to respond and the most effective administration regimen. An improved understanding of these issues would significantly optimize MSC treatment and help clinicians to better define the role of MSCs in the armamentarium of GvHD therapy.

In this study, we present the retrospective analysis of a cohort of 60 steroid‐resistant aGvHD patients treated with bone marrow‐derived MSCs at several Centres in the UK between May 2008 and December 2014. MSCs were manufactured at Imperial College Healthcare NHS Trust and administered for compassionate use [according to Regulation (EC) No 1394/2007]. aGvHD was biopsy proven in 45 patients, whilst in the remaining patients the diagnosis was based on clinical features after excluding alternative causes. Patients were considered to be steroid‐refractory when they failed to respond to high‐dose methylprednisolone (≥2 mg/kg body‐weight) after 6 days or if GvHD progressed after 3 days. Detailed demographics of patients are summarized in Table SI. Response to MSC treatment was assessed 1 week after administration. In accordance with previous experiences (Resnick *et al*, 2013; von Dalowski *et al*, 2016), responses were defined as complete (CR) in the event of complete disappearance of all symptoms and signs of the disease in all organs or partial (PR) when an improvement of at least 50% was observed in at least one organ affected by aGvHD. Stable or progressive disease were classified as no response (NR). Informed consent was obtained from all patients in accordance with the local ethics committee requirements. Data were analysed as of last data collection in June 2015. Additional details are available in the Data S1.

The median time from HSCT to MSC treatment was 62 days (range: 12–929 days), the median time from GvHD diagnosis to MSCs was 60 days (range: 11–905 days). Thirty‐four patients received 1 MSC dose, while 16, 6 and 1 patients were treated with 2, 3 and 4 doses, respectively. The median dose of MSCs was 2·6 × 10^6^/kg body‐weight per infusion (range: 0·6–15·6 × 10^6^/kg body‐weight). No significant adverse reactions were observed.

We selected to assess clinical responses 1 week after the first dose to obtain an early predictor of clinical outcome. Overall, 32 patients (53%) responded to MSCs, with 1 (1·6%) and 31 (51·6%) patients achieving CR or PR, respectively. Amongst patients who received multiples doses, in most cases subsequent doses did not change the type of response obtained after the first dose, with the exception of two patients. One patient responded to the first dose with a PR, received a second one but relapsed a week after. The second patient achieved PR after the first dose and CR after the second.

The estimated median overall survival (OS) of all patients was 104 days [95% confidence interval (CI): 0–215 days] (Fig 1A), with a median follow‐up of 741 days for patients alive (range 461–2521 days). Response to MSCs had a major impact on OS, with a longer estimated OS in responding patients compared with non‐responders (Fig 1B). We evaluated the association between OS and gender, age, pre‐MSC therapy, interval from HSCT or aGvHD diagnosis to MSC treatment, grade of aGvHD, organ involved and the response to MSCs. By using both univariate and multivariate analysis, we found that the presence of any kind of response (CR or PR) assessed after 1 week from the first MSC infusion and the organ affected by GvHD were strong predictors for survival (Table SII).

**Figure 1:**
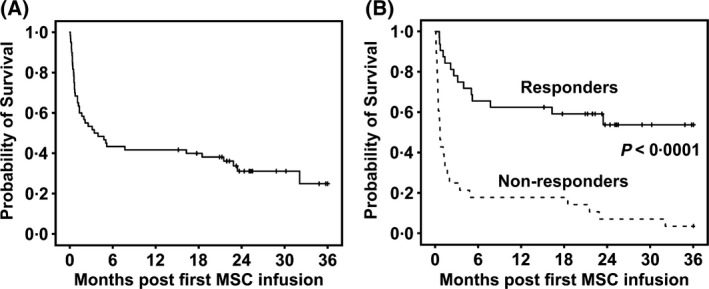
Probability of survival following MSC treatment. (A) Probability of survival of the whole cohort of patients. (B) Probability of survival of responders and non‐responders. log‐rank test, *P* < 0.0001; hazard ratio: 0·2. Median survival in responders was not reached, while it was 20 days (95% CI 11–29 days) in non‐responders. Overall survival estimates start from mesenchymal stromal cell (MSC) infusion.

We investigated whether we could identify factors associated with clinical responses. We found that patient gender, pre‐MSC therapy, interval from HSCT or aGvHD diagnosis to MSC treatment and grade of aGvHD did not affect clinical responses (Table SII). Conversely, organ involvement, age at HSCT and the dose of MSC infused were significantly associated with the response rate to MSC infusions (Table SII). The proportion of responders was 67% (*n* = 28) amongst patients with involvement of gut, skin or both, but only 22% (*n* = 4) amongst those with liver involvement (alone or in combination with skin and/or gut). Patients younger than 20 years fared better, with 88% (*n* = 15) of them responding to MSCs. Conversely, only 30% (*n* = 7) and 43% (*n* = 6) of those aged 20–50 years or older than 50 years responded, respectively. Lastly, higher response rates (77%, *n* = 13) were observed in patients receiving MSC doses >3·0 × 10^6^/kg compared with patients receiving 1·5–3·0 × 10^6^/kg (56%, *n* = 18) or <1·5 × 10^6^/kg (9%, *n* = 1) (Table SII). All these 3 factors remained significant in multivariate logistic regression analysis (Table 1).

**Table 1:** Multivariate logistic regression analysis for disease response

	*N*	Odds ratio (95% CI)	*P*
Patient age, years			
<20	17	1·00	
20–50	23	0·10 (0·02–0·72)	0·022
>50	14	0·45 (0·05–4·66)	0·46
aGvHD organ, *n*			
Skin or Gut or Skin + Gut	42	1·00	
Other	18	0·10 (0·01–0·78)	0·028
MSC dose, ×10^6^/kg body‐weight			
<1·5	11	1·00	
1·5–3·0	32	6·90 (0·55–86·50)	0·14
>3·0	17	28·22 (1·70–477·04)	0·021

aGvHD, acute graft‐versus‐host disease; CI, confidence interval; MSC, mesenchymal stromal cell.

Our data demonstrate that clinical responses at 1 week after MSC infusion can be considered an early predictor of clinical outcome. Such an approach is in contrast with the current practice of evaluating responses at 28 days (Resnick *et al*, 2013; von Dalowski *et al*, 2016) and might understate the rate and magnitude of clinical responses. In fact, the rate of CR in our cohort is very low compared to other studies (Le Blanc *et al*, 2008; Sánchez‐Guijo *et al*, 2014). In our study we could not document whether our responding patients eventually achieved CR at later time points, because we were unable to retrieve consistent data after day 7. However, the obtainment of any kind of response (either PR or CR) was sufficient to affect OS. This is consistent with previous studies in patients who, like ours, were refractory to several lines of treatment (Resnick *et al*, 2013; Kurtzberg *et al*, 2014; von Dalowski *et al*, 2016; Muroi *et al*, 2016; Dotoli *et al*, 2017; Servais *et al*, 2018). In contrast, other groups have reported that, when MSCs were administered soon after steroids, only CR are associated with improved OS (Le Blanc *et al*, 2008; Kebriaei *et al*, 2009; Sánchez‐Guijo *et al*, 2014). The results of a very recent study seem to explain the differences and reconcile the inconsistency. Although patients who have been heavily pre‐treated before MSC infusion achieve CR less frequently than those who receive MSCs as second line, the OS of the two groups does not differ, regardless of the response achieved (CR or PR) (Bader *et al*, 2018).

An early assessment of the response has therefore the advantage of providing crucial information to enable prompt management of alternative approaches both in clinical practice and in the context of clinical trials.

Because of the retrospective nature of the study, the dose range of MSCs was large, thereby allowing us to identify a significant association between higher doses and a positive response. This observation is in contrast with other reports (Ball *et al*, 2013; von Dalowski *et al*, 2016) but the discrepancy could be ascribed to the fact that, in those studies, the dose ranges were too narrow and the number of patients too small to identify any correlation between response and dose. Our data have been confirmed by a recent multicentre prospective study, in which patients who received doses of 3–4x10^6^/kg body‐weight had better responses and longer survival rates than those who received 1–2 × 10^6^/kg body‐weight (Servais *et al*, 2018). Our study confirms that patient age and the affected organ significantly affect responses to MSCs (Resnick *et al*, 2013; Sánchez‐Guijo *et al*, 2014). In contrast, we could not find any association between GvHD grade (Resnick *et al*, 2013) or time from GvHD to MSCs (Ball *et al*, 2013) and response to MSCs.

In summary, our data strengthen the role of the MSC recipient (Galleu *et al*, 2017) rather than the one of MSC donor or source in predicting clinical responses. This observation is supported by the findings of other groups (Le Blanc *et al*, 2008; Kebriaei *et al*, 2009; von Dalowski *et al*, 2016), whereby when MSCs from the same donor were used to treat several patients, only a proportion of them achieved remission. Such a perspective suggests that the variability in MSC manufacturing bears a limited impact (Trento *et al*, 2018). Overall, these findings provide an innovative angle that could be harnessed to improve the complex design of future clinical studies for the treatment of GvHD with MSCs.

## Authorship Contributions

A.G. and S.D. collected and elaborated the data; R.S. and A.G. performed the statistical analysis; A.G. analysed the results and prepared the figures. D.M., R.W., D.M., D.R., K. O., E.K., E.T., M.S., P.S., S.L., A.B., A.P., J.A.S., A.V., B.K., H.H., J.F.A. and F.D. provided the data on patients; S.L. provided clinical grade MSCs, A.G. and F.D. wrote the original draft of the manuscript; all authors contributed to the final version of the manuscript.

## Conflict of Interest Disclosures

Since 2016, J.A.S. has been Chair of NHS England Specialized Commissioning Clinical Reference Group for Blood and Marrow Transplantation. The other authors declare no competing financial interests.

## Supporting information


**Data S1.** Materials and methods.
**Table SI.** Patients’ characteristics.
**Table SII.** Univariate analyses of probabilities of survival and disease response.
