# Supplementary material for: Mesenchymal stromal cells for acute graft‐versus‐host disease: response at 1 week predicts probability of survival
Source: Br J Haematol. 2019 Jan 13;185(1):89–92. doi: 10.1111/bjh.15749 (PMC6916615; doi:10.1111/bjh.15749)
Supplement: Supplementary file 1 — Data S1. Materials and methods. Table SI. Patients’ characteristics. Table SII. Univariate analyses of probabilities of survival and disease response. [file BJH-185-89-s001.docx]

**Supporting information**

**Mesenchymal Stromal Cells for acute Graft versus Host Disease: response at one week predicts probability of survival**

Antonio Galleu^1,2^, Dragana Milojkovic^3^, Simona Deplano^3^, Richard Szydlo^4^, Sandra Loaiza^3^, Robert Wynn^5^, David I. Marks^6^, Deborah Richardson^7^, Kim Orchard^7^, Edward Kanfer^3^, Eleni Tholouli^5^, Muhammad Saif^5^, Ponni Sivaprakasam^6^, Sarah Lawson^8^, Adrian Bloor^9^, Antonio Pagliuca^10^, Varun Mehra^10^, John A. Snowden^11^, Ajay Vora^12^, Bhuvan Kishore^13^, Hannah Hunter^14^, Jane Apperley^3,4^*, and Francesco Dazzi^1,2,3^*.

^1^School of Cancer and Pharmaceutical Sciences King’s College London, London, UK. ^2^King’s Health Partners Cancer Research UK Centre, ^3^Imperial College Healthcare NHS Trust, London, UK, ^4^Imperial College London, London, UK, ^5^Central Manchester University Hospital, Manchester, UK, ^6^University Hospitals Bristol, Bristol, UK, ^7^University Hospital Southampton, Southampton, UK, ^8^Birmingham Women’s and Children’s Hospitals, Birmingham, UK, ^9^The Christie NHS Foundation Trust, Manchester, UK, ^10^King’s College Hospital NHS Trust, London, UK, ^11^Sheffield Teaching Hospitals NHS Foundation Trust, Sheffield, UK, ^12^Sheffield Children’s Hospital, Sheffield, UK, ^13^Heart of England NHS Foundation Trust, Birmingham, UK, ^14^Plymouth Hospitals NHS Trust, Plymouth, UK.

*Equally contributed to the study

**Supplementary Materials and Methods**

Sixty patients affected by steroid resistant aGvHD were treated with MSCs at the following UK centers: King’s College Hospital NHS Trust, Imperial College Healthcare NHS Trust, London, Central Manchester University Hospital, Manchester, University Hospitals Bristol, Bristol, University Hospital Southampton, Southampton, Birmingham Women’s and Children’s Hospitals, Birmingham, The Christie NHS Foundation Trust, Manchester, London, Royal Hallamshire Hospital, Sheffield, Sheffield Children’s Hospital, Sheffield, Heart of England NHS Foundation Trust, Birmingham and Plymouth Hospitals NHS Trust, Plymouth. Individual participant data will not be shared.

Clinical grade human bone-marrow derived MSCs were generated from bone marrow aspirates collected from the iliac crest of 2 healthy donors. Briefly, 2 ml of BM aspirate were collected in a tube with 100 µl preservative-free heparin. The cells were plated within 24 hours at a density of 10-25 million/636 cm^2^ by using alpha modified Eagle’s medium (ThermoFisher Scientific), conservative-free heparin (1 UI/ml) (Wockhardt UK Limited) and 5% platelet lysate and then incubated for 3 days at 37 °C and 5% CO_2_ ambience. Non-adherent cells were discarded by washing with phosphate buffered saline (ThermoFisher Scientific). When cell confluence of 90-100% was achieved cells were detached with Trypsin-EDTA (0.05% trypsin, 0.5γ mM EDTA•4Na) (ThermoFisher Scientific) and reseeded at a density of 5000 cells/cm^2^. Released criteria were based on positivity (>80%) for CD105, CD90, CD73, negativity (<2%) for CD3, CD14, CD19, CD31, CD45. All MSCs were used by passage three. All MSCs were manufactured at the John Goldman Centre for Cellular Therapy Imperial College Healthcare NHS Trust.

Statistics: Probabilities of survival were estimated using the Kaplan-Meier method. The associations of patient, disease and treatment factors with survival and disease response were investigated using the log-rank test, chi-squared test **or chi-squared trend test**, respectively.

P-values less than 5% (p<.05, two-sided) was considered statistically significant. SPSS version 25 (IBM Corp. Released 2017. IBM SPSS Statistics for Windows, Version 25.0. Armonk, NY: IBM Corp.) was utilized for all calculations.

**Table SI. Patients’ characteristics**

| **Total, number** | 60 |
| --- | --- |
| **Age, years** |  |
| Median (Range) | 40 (4mo-68) |
| **Sex, n** |  |
| Male | 21 |
| Female | 39 |
| **Disease, n** |  |
| AML | 14 |
| ALL | 10 |
| CML | 10 |
| MDS/MPNs | 7 |
| CLL | 1 |
| NHL/HL/MM/Other Lymphomas | 11 |
| Others | 7 |
| **Time from HSCT to MSC treatment, days** |  |
| Median (range) | 62 (12-929) |
| **Time from aGvHD to MSC treatment, days** |  |
| Median (range) | 60 (11-905) |
| **GvHD treatment before MSC infusion, number** |  |
| MEP Alone | 13 |
| MEP in combination with other drugs | 45 |
| Other drug combinations not including MEP | 2 |
| CSA | 17 |
| MMF | 30 |
| Anti-TNFα (Infliximab, Etanercept) | 18 |
| Tacrolimus | 8 |
| MTX | 3 |
| Anti-CD25 (Basiliximab, Daclizumab) | 1 |
| ECP | 3 |
| Anti-CD20 (Rituximab) | 1 |
| Anti-CD52 (Alemtuzumab) | 3 |
| ATG | 1 |
| **GvHD grade, number** |  |
| I-II | 5 |
| III-IV | 55 |
| **Biopsy Proven, number** |  |
| Yes | 46 |
| No | 14 |

**aGvHD:** acute Graft versus Host disease, **AML:** Acute Myeloid Leukemia, **ALL:** Acute Lymphoblastic Leukemia, **ATG:** anti-thymocyte globulin, **CML:** Chronic Myeloid Leukemia, **CLL:** Chronic Lymphocytic Leukemia, **CSA:** Cyclosporin, **ECP:** Extracorporeal photopheresis**, HSCT:** Hematopoietic Stem Cell Transplant, **MDS/MPN:** Myelodisplastic Syndrome/Myeloproliferative Neoplasms, **MEP:** Methylprednisolone, **MMF:** Mycophenolate, **MSC:** Mesenchymal Stromal Cells, **MTX:** Methotrexate**, NHL/HL/MM:** Non-Hodgkin Lymphoma/Hodgkin Lymphoma/Multiple Myeloma, **TNFα:** Tumor Necrosis Factor α.

**Table SII. Univariate analyses of probabilities of survival and disease response.**

|  | **N** | **Median Survival, months (95%CI)** | ***P*** | **Response, %** | ***P*** |
| --- | --- | --- | --- | --- | --- |
| Overall, number | **60** | **3.4 (0-7.8)** | **-** | **32 (53%)** | **-** |
| Patient Gender, number  Male  Female | **21**  **39** | **2.6 (0-7.3)**  **7.7 (0-27.1)** | ***0.23*** | **12 (57%)**  **20 (51%)** | ***0.66*** |
| Patient age, years  <20  20-50  >50 | **17**  **23**  **14** | **16.3 (NC)**  **1.3 (0-7.1)**  **1.7 (0.5–2.8)** | ***0.11*** | **15 (88%)**  **7 (30%)**  **6 (43%)** | ***0.008*** |
| aGvHD treatment, number  MEP alone  MEP +1 other  MEP +2 or more | **12**  **13**  **30** | **4.8 (0-27)**  **5.0 (0.6-9.5)**  **2.1 (0-5.8)** | ***0.96*** | **4 (33%)**  **8 (61%)**  **20 (67%)** | ***0.14*** |
| Time from HSCT to MSCs, days  <60  >59 | **26**  **28** | **1.9 (0-4.3)**  **16.3 (0-39)** | ***0.23*** | **14 (54%)**  **14 (50%)** | ***0.78*** |
| Time from GvHD to MSCs, days  <60  >59 | **28**  **22** | **3.1 (0-6.7)**  **1.7 (0-5.0)** | ***0.82*** | **16 (57%)**  **8 (36%)** | ***0.14*** |
| aGvHD grade, number  2  3  4 | **5**  **25**  **29** | **22.9 (0-37)**  **2.3(0-9.0)**  **2.7 (0-5.5)** | ***0.46*** | **1 (20%)**  **15 (60%)**  **16 (55%)** | ***0.42*** |
| aGvHD organ  Skin or Gut or Skin + Gut  Other | **42**  **18** | **16.3 (0-37.0)**  **0.6 (0.3-1.0)** | ***0.008*** | **28 (67%)**  **4 (22%)** | ***0.002*** |

| MSC dose, x10^6^/Kg body-weight  <1.5  1.5-3.0  >3.0 | 11  32  17 | 2.2 (0-4.7)  3.9 (0-11.9)  5.0 (0-10.1) | *0.62* | 1 (9%)  18 (56%)  13 (77%) | *0.001* |
| --- | --- | --- | --- | --- | --- |
| Response to MSCs  Yes  No | 32  28 | Not achieved  0.6 (0.4-1.0) | *<0.001* |  |  |

**aGvHD:** acute Graft versus Host disease, **HSCT**: Hematopoietic Stem Cell Transplant**, MEP:** Methylprednisolone, **MSCs:** Mesenchymal Stromal Cells.
